# Evaluation of unsulfated biotechnological chondroitin in a knee osteoarthritis mouse model as a potential novel functional ingredient in nutraceuticals and pharmaceuticals

**DOI:** 10.3389/fbioe.2022.934997

**Published:** 2022-11-17

**Authors:** Donatella Cimini, Serena Boccella, Alberto Alfano, Antonietta Stellavato, Salvatore Paino, Chiara Schiraldi, Francesca Guida, Michela Perrone, Maria Donniacuo, Virginia Tirino, Vincenzo Desiderio, Barbara Rinaldi

**Affiliations:** ^1^ Department of Environmental, Biological and Pharmaceutical Sciences and Technologies, University of Campania L. Vanvitelli, Naples, Italy; ^2^ Department of Experimental Medicine, Section of Biotechnology, Medical Histology and Molecular Biology Naples, University of Campania L. Vanvitelli, Naples, Italy

**Keywords:** osteoarthritis, chondroitin, mouse model, chondroitin sulfate, functional ingredient

## Abstract

Osteoarthritis is a very disabling disease that can be treated with both non-pharmacological and pharmacological approaches. In the last years, pharmaceutical-grade chondroitin sulfate (CS) and glucosamine emerged as symptomatic slow-acting molecules, effective in pain reduction and improved function in patients affected by osteoarthritis. CS is a sulfated glycosaminoglycan that is currently produced mainly by extraction from animal tissues, and it is commercialized as a pharmaceutical-grade ingredient and/or food supplement. However, public concern on animal product derivatives has prompted the search for alternative non-extractive production routes. Thus, different approaches were established to obtain animal-free natural identical CS. On the other hand, the unsulfated chondroitin, which can be obtained *via* biotechnological processes, demonstrated promising anti-inflammatory properties *in vitro*, in chondrocytes isolated from osteoarthritic patients. Therefore, the aim of this study was to explore the potential of chondroitin, with respect to the better-known CS, in an *in vivo* mouse model of knee osteoarthritis. Results indicate that the treatment with biotechnological chondroitin (BC), similarly to CS, significantly reduced the severity of mechanical allodynia in an MIA-induced osteoarthritic mouse model. Decreased cartilage damage and a reduction of inflammation- and pain-related biochemical markers were also observed. Overall, our data support a beneficial activity of biotechnological unsulfated chondroitin in the osteoarthritis model tested, thus suggesting BC as a potential functional ingredient in pharmaceuticals and nutraceuticals with the advantage of avoiding animal tissue extraction.

## 1 Introduction

Osteoarthritis (OA) is one of the major causes of disability in millions of people worldwide. OA is a very common degenerative joint disease characterized by multifactorial progressive breakdown of the articular cartilage, which has an extremely limited capacity of intrinsic repair. A recent population-based study established that over 600 million individuals suffered from knee OA in 2020 (Cui et al., 2020), with a growing incidence of the pathology also among younger people.

Articular cartilage is composed of highly specialized and metabolically active cells called chondrocytes that are interspersed in the extracellular matrix (ECM), a dense and organized structure that contains proteins and glycosaminoglycans (GAGs) including hyaluronic acid (HA) and chondroitin sulfate (CS) (Fox et al., 2009). To avoid side effects caused by long-term drug therapies, among non-pharmacological options for the treatment of OA, the administration of chondroitin sulfate (frequently combined to glucosamine) is also recommended. The use of CS was in fact shown to be beneficial in reducing pain, improving function, and even preventing or reverting the structural degeneration of the affected tissues (McCarty et al., 2000; Uebelhart et al., 2004; David-Raoudi et al., 2009; Kahan et al., 2009; Kwan Tat et al., 2010). Indeed, the degraded cartilage of osteoarthritic knees presents lower concentrations and length of the chains of glycosaminoglycans, like CS and hyaluronic acid, and downregulation of chondroitin sulfate glycosyltransferase genes (enzymes necessary for CS synthesis) (Ishimaru et al., 2014). In fact, in addition to hyaluronic acid being the major responsible factor for the mechanical and elastic properties of articular cartilage, CS also boosts the synthesis of new joint cartilage (Stellavato et al., 2016).

When CS is marketed as a pharmaceutical-grade ingredient, good manufacturing practices (GMPs) ensure product quality; however, a growing number of CS-based products are commercialized as food supplements, and in this case the production processes do not undergo the strict regulatory controls applied for medicinal drugs. This often leads to the marketing of poorly characterized, heterogeneous, and possibly less bioactive products. Indeed, CS is still manufactured by extraction from animal tissues, and it was observed that the type and purity of the source material greatly affect results of scientific studies and clinical trials (Malavaki et al., 2008; Kwan Tat et al., 2010). Moreover, ethical and religious public concerns related to animal-derived products can limit growth of the CS market, thus opening the way to safer and potentially unlimited biotechnological production routes. From this aspect, numerous microorganisms were investigated up to date as alternative cell factories for the fermentation process setup (Cimini et al., 2018). Some pathogenic bacteria (e.g., *Escherichia coli* K4, *Pasteurella multocida* type F, and *Avibacterium paragallinarum*) naturally produce a polysaccharide with a carbon backbone that is identical to unsulfated chondroitin, and that can be chemically or enzymatically converted to CS; moreover, also other strains (e.g., *Bacillus subtilis* and *E. coli* K12) were used as hosts in unsulfated chondroitin production (Cimini et al., 2018). Animal-free natural identical CS was previously obtained by either combining fermentation and chemical sulfation (Bedini et al., 2011) or by using a one-step, *in vivo*, microbial synthesis approach (Badri et al., 2021).

Previous studies (Shipp and Hsieh-Wilson, 2007; Sirko et al., 2007; Izumikawa et al., 2015; Djerbal et al., 2017; Zhu et al., 2018) demonstrated that the sulfation pattern defines the binding specificities of different CS, thereby regulating diverse cellular processes and providing CS with different roles and properties (e.g., anti-obesity, anti-diabetic, and immuno-modulatory activities).

Interestingly, unsulfated highly pure biotechnological chondroitin (BC) also demonstrated compelling properties *in vitro*. BC was more effective than commercial CS in reducing the inflammatory response induced by IL-1β in chondrocytes and in preserving the chondrogenic phenotype (Stellavato et al., 2016). This property was also confirmed by a recent study showing that HA/BC gels induced the differentiation of chondrocytes stimulating the production of type II collagen (Alessio et al., 2021). Moreover, the treatment of damaged human articular chondrocytes, isolated from osteoarthritic patients, with BC singly or in combination with high molecular weight HA, reduced the expression of crucial inflammatory mediators endorsing the anti-inflammatory potential of BC even beyond CS (Vassallo et al., 2021). Although results from *in vitro* models are promising, additional experiments are necessary to ascertain the efficacy of BC in the treatment of OA *in vivo*.

The present work describes for the first time, to the best of our knowledge, the use of biotechnological unsulfated chondroitin in a mouse model of knee OA. In particular, the beneficial effects of a BC-repeated treatment were evaluated by measuring the pain threshold and pain-related pro-inflammatory mediators in an intra-articular monoiodoacetate (MIA) model. Moreover, the contribution of the immune system through the analysis of the percentage of a specialized subpopulation of T regulatory cells (Tregs) in OA animals was investigated.

## 2 Materials and methods

### 2.1 Materials

CS (chondroitin 4, 6 sulfate) used in this study is commercially available and extracted from fish cartilage (Condrosulf, IBSA, Lugano, Switzerland), particularly from mako shark, brown shark, *Carcharinus menisorrah*, and sorrah shark that are not protected species.

### 2.2 Production and purification of BC

The strain EcK4r3 (Cimini et al., 2013) was grown on a 15-L Biostat C bioreactor (Sartorius Stedim, Germany) under fed-batch conditions according to a previously developed protocol (D’ambrosio et al., 2020; De Rosa et al., 2010). After 23 h of growth, the supernatant containing the fructosylated chondroitin capsular polysaccharide was recovered by centrifugation, then microfiltered and treated with proteases (10 U/L, Sigma-Aldrich, Missouri, United States) to reduce protein contamination. The supernatant was concentrated and diafiltered by tangential flow filtration on a UniFlux-10, (GE Healthcare, Illinois, United States) on 30-kDa polyethersulfone membranes (GE Healthcare, Illinois, United States) to eliminate low molecular weight contaminants and hydrolyzed at 90°, pH 2.8 ± 0.1 for 60 min to obtain the defructosylation of the polymer and a resulting backbone composed of β-1,3-linked N-acetylgalactosamine and glucuronic acid that is identical to that of CS. A second UF step was next conducted on 5-kDa membranes. The retentate was precipitated with 3 V of cold ethanol 96% (v/v) and a conductivity of about 13–15 mS/cm by letting aggregates sediment o/n. The obtained precipitate was re-dissolved in deionized water and treated with the charcoal powder (Sigma-Aldrich, Italy) in batch for both decolorization and reduction of endotoxin content. Repeated precipitations were performed to obtain purity higher than 96% on a dry basis as evaluated by capillary electrophoresis and SEC-TDA, with the endotoxin content lower than 1 EU/mg. The powder obtained was characterized following the European Pharmacopoeia methods as subsequently described. The gel-clotting method and the kinetic LAL (limulus amebocyte lysate) assay (the bacterial endotoxin test, 2.6.14 European Pharmacopoeia. 01/2005:20614) (Endosafe, Charles River, United States) were used to determine the endotoxin content in the final powder (Ding and Ho, 2001). The latter was characterized by SEC-TDA and capillary electrophoresis (Restaino et al., 2009). The absence of DNA and protein contaminants was verified according to the pharmacopoeia (Eu.ph.2.2.25). The total viable aerobic count was also performed (Eu.Ph.2.6.12) to ascertain the absence of microbial contamination within pharmacopoeia indications.

### 2.3 SEC-TDA molecular weight determination

Molecular weight analyses of the purified chondroitin and extracted CS were performed as previously described (Restaino et al., 2019) by a high-performance size-exclusion chromatographic system (Malvern, United Kingdom), equipped with a triple detector array module including a refractive index detector (RI), a four-bridge viscosimeter (VIS), a laser detector (LS) made of a right-angle light scattering (RALS) detector and a low-angle light scattering (LALS) detector, and two gel-permeation columns (TSK-GEL GMPWXL, 7.8 × 30.0 cm, Tosoh Bioscience, Italy). Polyethylene oxide (PEO) was used as a standard for the instrument calibration (22 kDa PolyCAL, Viscotek, Malvern). The average molecular weight, polydispersity index (Mw/Mn), and intrinsic viscosity (IV) were determined by all the detector signals applying the equations reported by the manufacturer (data from Viscotek) and based on the dn/dc values of BC and CS equal to 0.155 and 0.146, respectively.

### 2.4 Animals

Male C57BL/6 mice (Charles River, Italy) of age 7–8 weeks weighing 18–20 g were housed three per cage under controlled illumination (12 h light/dark cycle; light on 6:00 a.m.) and standard environmental conditions (ambient temperature 20°C–22°C and humidity 55%–60%) for at least 1 week before the commencement of experiments. Mice chow and tap water were made available *ad libitum*. The Animal Ethics Committee of the University of Campania “L. Vanvitelli” approved the experimental procedures. Animal care was in compliance with Italian (D.L. 116/92) and European Commission (O.J. of E.C. L358/1 18/12/86) regulations on the protection of laboratory animals. All efforts were made to reduce both animal numbers and suffering during the experiments.

### 2.5 Induction of osteoarthritis

Osteoarthritis was induced by a single injection into the knee joint by monoiodoacetate (MIA) at a dose of 1 mg/mouse. Mice were anesthetized with tribromoethanol (125 mg/kg) and placed on their back. To stabilize the injection site, the knee was held still, in a bent position, by placing the index finger under the knee joint and the thumb over the anterior surface of the ankle joint. To find the precise injection site, a 26 G needle attached to a syringe was slid horizontally along the knee (so as not to puncture the skin with the tip) until the space under the patella was found. A gentle pressure was then applied to mark the area, and the needle and syringe were lifted vertically for injection. The needle was inserted into the marked area, through the patellar tendon, perpendicular to the tibia. Using the thumb as a guide, MIA was injected superficially at the entry site. After injection, the knee was massaged to ensure even distribution of the solution, and the mice were placed back in their cages to allow for recovery from the anesthesia. Sham mice received vehicle instead of MIA. A total number of 36 mice were divided into six experimental groups: sham/veh, MIA/veh, MIA/CS (200 and 400 mg/kg), and MIA/BC (200 and 400 mg/kg). The von Frey test was performed at the baseline and at different time points after MIA injection (7, 14, 21, and 28 days). After behavioral testing, the mice were sacrificed for biochemical evaluations.

### 2.6 Drugs

Sodium iodoacetate was purchased from Sigma-Aldrich (I-2512-25G). Chondroitin sulfate and biotechnological chondroitin at the doses of 200 and 400 mg/kg or vehicle (saline) were administered *via* gavage starting from day 7 post induction for three weeks. The doses used were chosen according to previous studies (Fernández-Martin et al., 2021).

### 2.7 Experimental design

**FIGURE 1:**
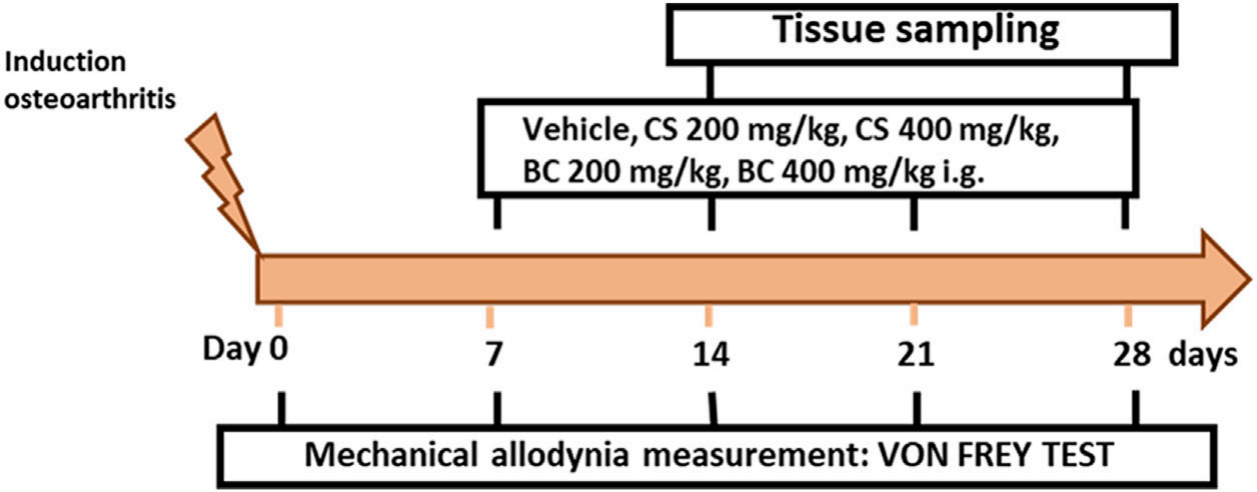
Timeline of the experimental procedure of osteoarthritis induction and related behavioral characterization in the presence of vehicle, CS, or BC treatment. On day 0, after mechanical allodynia basal-level measurements, mice received a single intra-articular injection of vehicle or monoiodoacetate (1 mg/mouse). Chondroitin sulfate (CS) or biotechnological (BC) and saline (vehicle) were administered daily for three consecutive weeks starting from day 7 after MIA administration.

A total number of 36 mice were divided into six experimental groups: sham/veh (*n* = 6), MIA/veh (*n* = 6), MIA/CS 200 mg/kg (*n* = 6), MIA/CS 400 mg/kg (*n* = 6), MIA/BC 200 mg/kg (*n* = 6), and MIA/BC 400 mg/kg (*n* = 6). The von Frey test was performed at different time points (0, 7, 14, 21, and 28 days) to test the effect of drugs on mechanical allodynia. At 14 and 28 days, mice were sacrificed for biochemical evaluations. The timeline of osteoarthritis induction, treatments, and behavioral characterization is given in Figure 1.

### 2.8 Mechanical allodynia

Mechanical allodynia was evaluated by a series of calibrated von Frey filaments (Stoelting, Wood Dale, IL, United States), ranging from 0.008 to 2 g. Animals were allowed to freely move in the compartment positioned on the surface of the wire mesh for about 1 h before the test. The von Frey filaments were applied in increasing order on the mid-plantar surface of the hind paw through the mesh floor. If using the filament thrice did not induce a reaction, the next filament with higher pressure was used. The behavioral responses (rapid withdrawal of the paw and licking or shaking of the paw, during the application or immediately after the removal of the filament) were expressed as the mean ± S.E.M. of the paw withdrawal threshold (PWT) in grams.

### 2.9 Histology

For histological analysis, the sham or arthritic paws were cut and fixed in phosphate-buffered paraformaldehyde solution followed by decalcification. They were dehydrated and embedded in OCT (Killik, Bio Optica), frozen in liquid nitrogen, and stored at −80°C. Joint sections (20 μm) obtained using a cryostat (Leica CM 3050S) were stained with hematoxylin and eosin histological staining protocol. Samples were also prepared for immunofluorescence (Vassallo et al., 2022). Briefly, the samples were permeabilized and then were incubated with a blocking solution. The primary antibody against COMP-2 (diluted 1:100; Abcam, Cambridge MA) was incubated overnight. After 16 h, the slices were washed in PBS and incubated with the FITC-conjugated goat anti-rabbit secondary antibody (diluted 1:100; Life Technologies, Milano, Italy) for 1 h. Nuclei were stained with 2′-(4-hydroxyphenyl)-5-(4-methyl-1-piperazinyl)-2,5′-bi-1H-benzimidazole trihydrochloride hydrate and bisbenzimide (Hoechst 0.5 mg/ml, Sigma-Aldrich, Milano, Italy), and cytoskeleton was stained with phalloidin (Life Technologies, Milano, Italy). Finally, the slices after sealing were observed on an Axiovert 200 (Zeiss) fluorescence microscope.

### 2.10 Evaluation of regulatory T lymphocytes

We evaluated the regulatory T lymphocyte (Treg) percentage in treated and untreated MIA mice after 30 days of treatment. The regulatory T lymphocytes showed the following phenotype: CD4^+^CD25^+^CD127low/-. Blood samples (200 μL) were collected in sterile EDTA vacutainers. Peripheral blood samples (100 μL) were incubated with a monoclonal antibody cocktail as follows: APC-H7-conjugated anti-CD4, APC-conjugated anti-CD25, FITC-conjugated anti-CD127, and PerCP-cy5.5-conjugated anti-CD45 for 30 min at 4°C. After incubation, the blood samples were treated with 2 ml FACS lysing solution (BD Biosciences) for 10 min at room temperature in dark and then washed with PBS, and they were acquired on a FACS Canto II flow cytometer and analyzed using DIVA software (BD Biosciences) version 8. One-way ANOVA was used to compare treated samples with an untreated control group. The graphs were generated using GraphPad Prism version 8.

### 2.11 Bio-Plex assay

To evaluate the anti-inflammatory effect of chondroitin sulfate (CS) and biotechnological unsulfated chondroitin (BC), a mouse model of MIA-induced knee osteoarthritis was used. Mice blood samples were collected after 14 and 28 days of treatment and centrifuged at 4,000 rpm for 10 min at 4°C to quantify cytokines in mice serum. Cytokines were assayed using multiplex biometric ELISA-based immunoassay, containing dyed microspheres conjugated with a monoclonal antibody specific for a target protein (Bio-Plex, Bio-Rad Lab, Milan, Italy). Specifically, pro-inflammatory and anti-inflammatory cytokines were quantified using the 8-plex immunoassay panel: GM-CSF, IFN-γ, IL-1β, IL-2, IL-4, IL-5, IL-10, and TNF-α. Each experiment was performed using four mice for the experimental point, and cytokine levels of all targets were determined using a Bio-Plex array reader (Luminex, Austin, TX). The analytic concentrations were calculated using a standard curve according to the manufacturer’s protocol.

## 3 Results

### 3.1 Purification and characterization of BC in comparison to CS

After fermentation, the purification procedure applied to the broth in this study was necessary to obtain unsulfated chondroitin with a high purity and low endotoxin content according to the pharmacopoeia and with similar characteristics to those of the commercial CS used in *in vivo* experiments. A complete characterization of the two samples, also regarding the eventual presence of microbial contaminants and of nucleic acids and proteins, thus fulfilling regulatory requirements, is reported in Table 1. Capillary electrophoresis and SEC-TDA analyses showed that BC and CS samples presented a high level of purity (>95%) and that the Mw and polydispersity of the two polymers were also rather similar (Table 1; Supplementary Figure S1). BC presented lower protein contamination than CS. The endotoxin content of BC demonstrated being suitable for oral administration of the active principle.

**TABLE 1:** Complete characterization of BC and CS.

Product description/analysis	BC	CS
Appearance	White or almost white powder	White or almost white powder
Sample purity (capillary electrophoresis)	95% ± 2%	99% ± 2%
Intrinsic viscosity at 25°C (SEC-TDA)	1.1 dl/g	0.9 dl/g
Mw (SEC-TDA)	35 ± 2 kDa	35 ± 2 kDa
Polydispersity Mw/Mn (SEC-TDA)	1.17	1.22
Proteins (Eu. Ph. 2.2.25)	<0.1%	1.4%
Water content	≤20%	10.2%
Bacterial endotoxins	≤1 EU/mg	≤1 EU/mg
Nucleic acids. Abs260 nm (solution 0.33% w/v)	≤0.01	≤0.01
Microbial contamination (TAMC, Eu. Ph. 2.6.12)	0 CFU/g	<5 CFU/g

### 3.2 Effect of BC or CS treatment on the mechanical allodynia in MIA-injected mice

**FIGURE 2:**
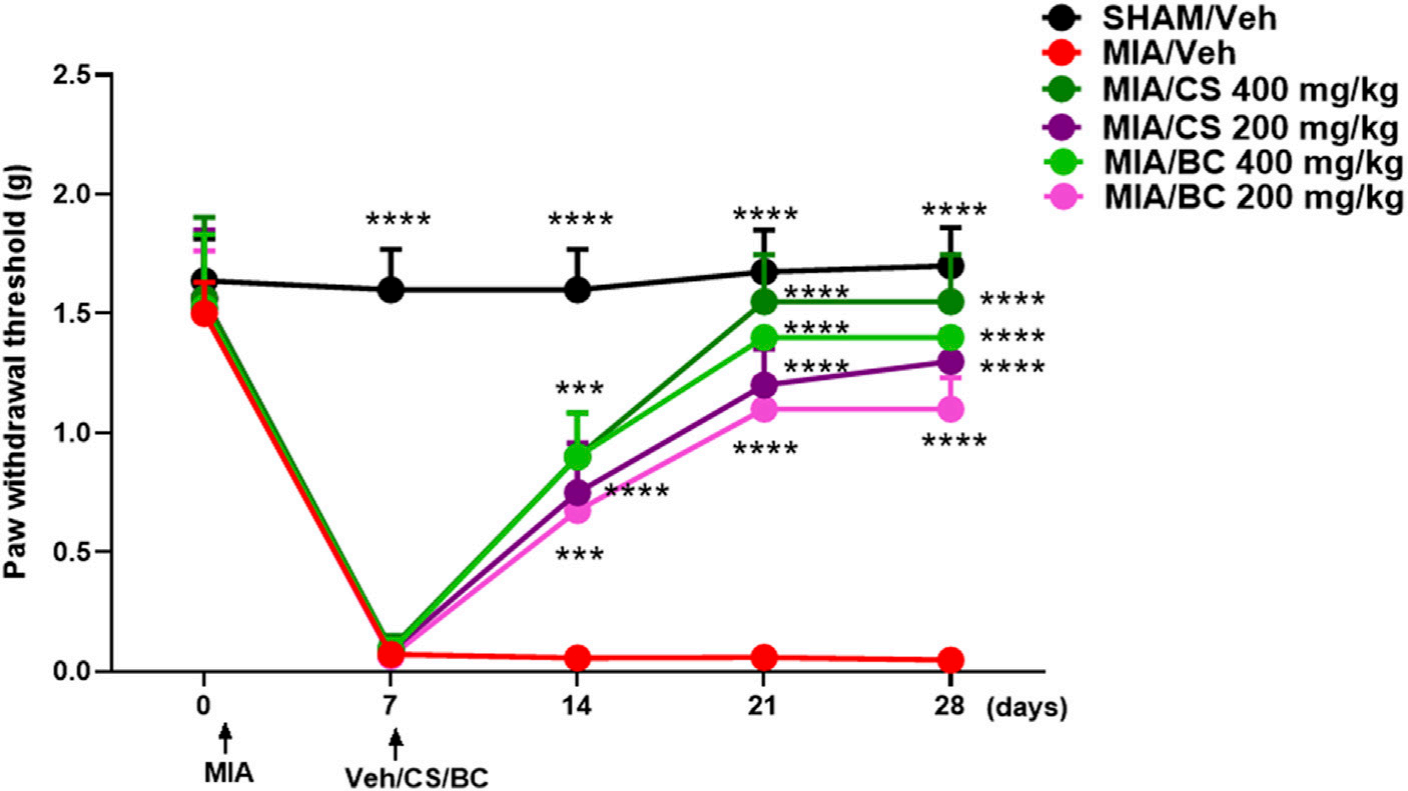
Effect of daily administration with CS or BC (at the doses of 200 and 400 mg/kg) or vehicle (saline) on the mechanical allodynia in MIA-injected mice. Time-dependent changes in the paw withdrawal response (g) in the sham group in MIA mice treated with vehicle or CS or BC at different doses. ***p < 0.001 and ****p < 0.0001 indicate significant differences vs. MIA/veh. Two-way ANOVA, followed by Tukey’s *post hoc* test.

MIA injection into the mouse knee triggers mechanical hypersensitivity in the ipsilateral hind paw that occurs in a biphasic manner in an early (0–10 days) and late (14–28 days) phase (Ogbonna et al., 2013). On the previous day before MIA injection, the basal withdrawal threshold values were recorded using the von Frey test. The development of mechanical allodynia was observed starting from day 3 or 7 after MIA injection (0.071 g ± 0.014), as compared to sham animals (1.6 g ± 0.060) (Figure 2 and Supplementary Figure S2). Sham mice experienced no changes in the withdrawal threshold until the end of evaluation. Daily treatment with CS (200 and 400 mg/kg) significantly reduced the osteoarthritis-induced mechanical allodynia at 7 (0.09625 g ± 0.019), 14 (0.9 g ± 0.065), 21 (1.55 g ± 0.069), and 28 (1.55 g ± 0.069) days post-MIA injection, as compared to vehicle (Figure 2 and Supplementary Figure S2). The effect was dose-dependent. In a similar fashion, BC restored the mechanical threshold at 7 (0.085 g ± 0.017), 14 (0.900 g ± 0.06), 21 (1.400 g ± 0.0001), and 28 (1.400 g ± 0.0001) days post-MIA injection, as compared to vehicle (Figure 2 and Supplementary Figure S2). No significant changes were observed between CS and BC (200 and 400 mg/kg) treatments in MIA-injected mice.

### 3.3 Effect of BC or CS treatment on cartilage alteration and on the expression of cartilage turnover markers

**FIGURE 3:**
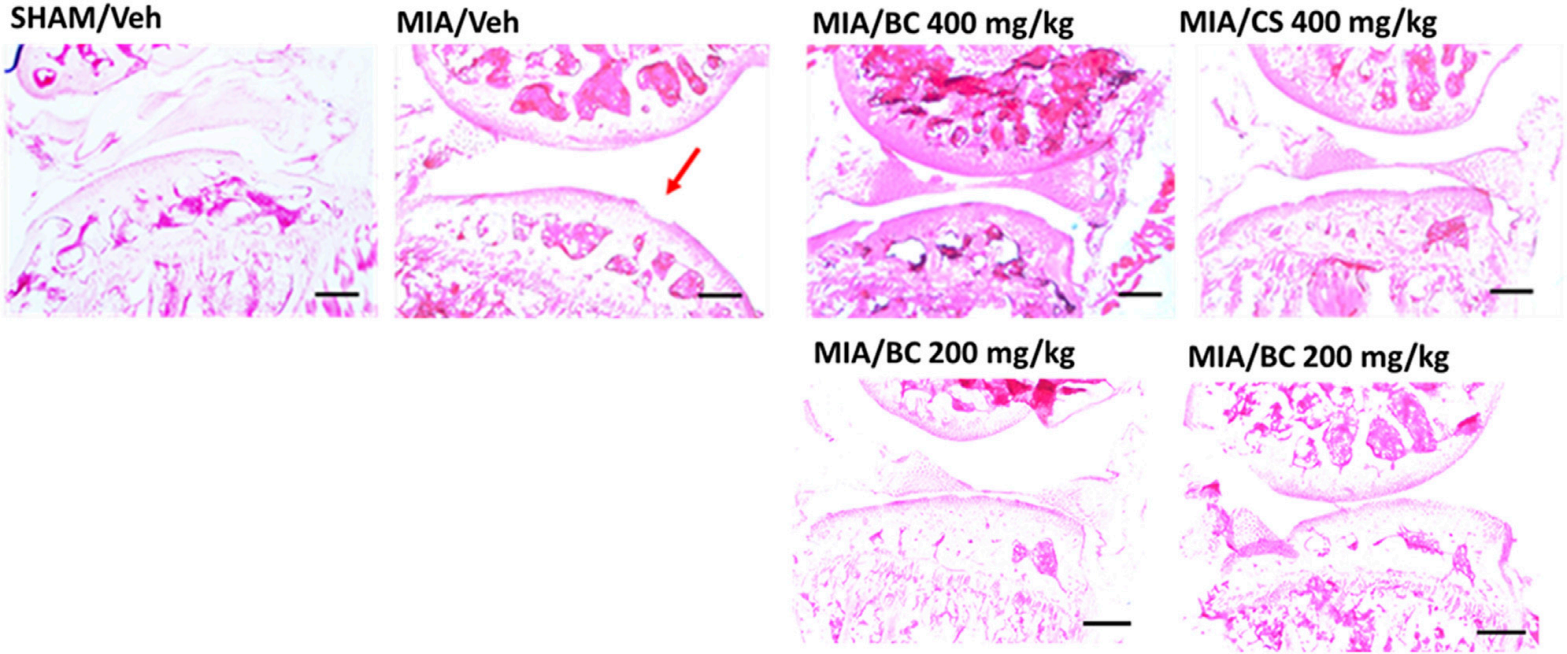
Histological analysis of MIA-treated mice. Hematoxylin and eosin staining of the representative mouse joint (*n* = 3 per group) of sham/veh, MIA/veh, MIA/CS (200 and 400 mg/kg), and MIA/BC (200 and 400 mg/kg) mice. Red arrow shows damaged areas. Scale bar = 40 µm.

Pathological changes induced by MIA share many common traits with those observed in human OA (Guzman et al., 2003), including loss of cartilage and alterations in the subchondral bone. Articular histological assessment of MIA mice 14 days post-induction indicates a focal area of cartilage loss as compared with that of the sham group. Interestingly, this effect appeared partially restored in BC- or CS (200 and 400 mg/kg)-treated MIA mice (Figure 3). According to previous studies (de Sousa Valente, 2019), 28 days post-injury, a partial recovery of bone damage was found (not shown).

**FIGURE 4:**
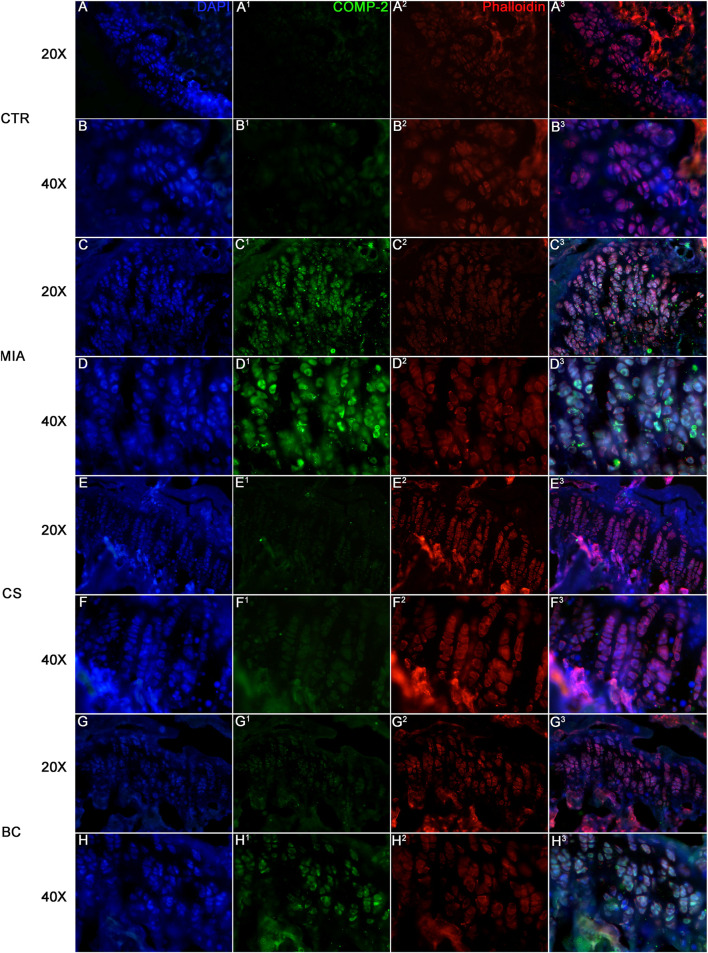
Immunofluorescence on tissue sections from mice knee joints. Sections were stained with DAPI for nuclei, phalloidin for cytoskeleton, and COMP. CTR is represented in figure **(A)** (20× magnification) and **(B)** (40× magnification), MIA in figure **(C)** (20× magnification) and **(D)** (40× magnification), CS in figure **(E)** (20× magnification) and **(F)** (40× magnification), and BC in figure **(G)** (20× magnification) and **(H)** (40× magnification). The expression of COMP is a marker of cartilage turnover. The images show how COMP is expressed in MIA groups of mice while it is almost absent in BC and CS groups.

The expression of cartilage oligomeric matrix protein (COMP-2) was assessed in all groups. COMP-2 is a glycoprotein that regulates the formation of type II collagen fibers in cartilage and their assembly. It is a cartilage turnover marker that has been demonstrated to rise in damaged cartilage and in the serum of OA patients (Petersen et al., 2010). The IF clearly demonstrates a strong expression of COMP-2 in the MIA group while being less expressed and thus not evidenced in IF images of the CTR, CS, and BC groups (Figure 4). These findings support the significance of chondroitin in cartilage physiological restoration and show that CS and BC have a comparable impact.

### 3.4 Effect of MIA, BC, or CS treatment on the percentage of T regulatory lymphocytes

To evaluate the effect of BC on inflammation, changes in the percentage of T regulatory cells were investigated in control and treated mice.

**FIGURE 5:**
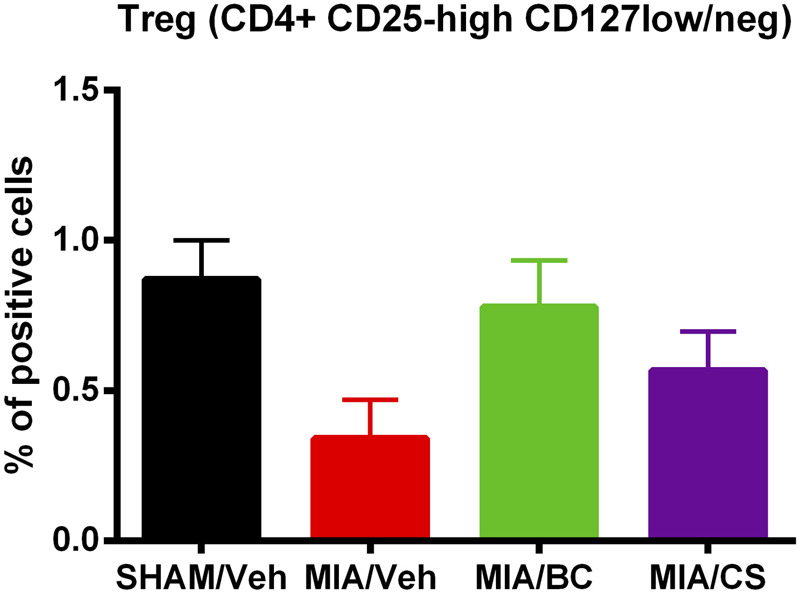
Analysis of the percentage of specialized subpopulation of T regulatory cells (Tregs) in sham and MIA-injected mice treated with BC or CS (200 and 400 mg/kg). The panel shows changes in the percentage of T CD4^+^ lymphocytes expressing CD25-high CD127-low/neg. Values are expressed as mean ± S.E.M. of measurements of at least three independently performed experiments. The two-way ANOVA test was employed, and *p* < 0.05 was considered statistically significant.

An increase in T regulatory cell percentage in treated mice with respect to MIA mice was observed. Although the use of BC increased levels of T regulatory lymphocytes more than CS (Figure 5), differences between CS- and BC (200 and 400 mg/kg)-treated groups were not statistically significant. These data indicate that CS and BC may induce Treg enrichment to promote inflammation suppression, as compared with the vehicle-treated MIA mice group, and the inflammation is not modulated by Treg.

### 3.5 Effect of BC on the expression of pro-inflammatory cytokines

**FIGURE 6:**
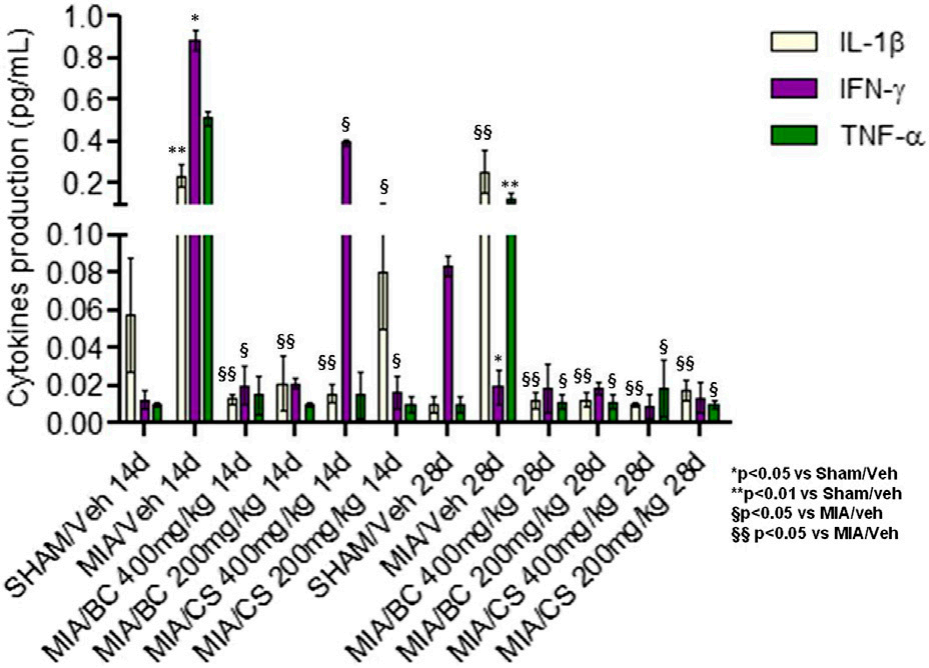
Cytokine production assay in mice serum. Significant protein levels in the control (sham treatment), in MIA, in BC, and in CS (tested at 200 and 400 mg/kg) are shown. The groups (BC and CS vs. MIA and vs. sham) are significantly different (§*p* < 0.05 and §§*p* < 0.01, and *p < 0.05 and **p < 0.01) according to Student’s *t*-test.

To evaluate how chondroitin-based treatments affected secreted biological mediators in MIA-induced knee osteoarthritis in mice, a multiplex assay was performed. Among the eight cytokines evaluated, the ones that were not statistically different from the controls are not shown. All the reported biological factors were significantly (*p < 0.05 and **p < 0.01) upregulated in MIA in comparison with untreated mice (sham). As shown in Figure 6, both CS and BC tested at 200 and 400 mg/kg proved effective on the reduction of three pro-inflammatory cytokines. Specifically, CS at 400 mg/kg was more effective than CS at 200 mg/kg in the modulation of IL-1β after 14 days of treatment. Also, BC resulted more effective at 400 mg/kg than 200 mg/kg on IL-1β and IFN-ƴ at 14 days (§§*p* < 0.01 and §*p* < 0.05), while, after 28 days of treatment for all three cytokines evaluated here, the two treatments similarly reduced the pro-inflammatory mediators.

## 4 Discussion

CS by itself or in combination with other active ingredients is currently used for the cure of OA. In particular, the use of CS as a food supplement and pharmaceutical has a long tradition. The registration as a pharmaceutical active principle, with specific functions, is, however, limited to a high product quality and purity approved by the European regulatory office and to products derived by extraction from porcine, bovine, and fish cartilage. In fact, depending on the MW and structure heterogeneity, and especially on the purity/quality ratio, the biological effect of extractive CS and thus the clinical outcome greatly vary. Accordingly, recent research efforts regarded the identification of analytical techniques to characterize samples from different raw materials and identify potential contaminants or even adulterants (Restaino et al., 2019).

The use of biotechnological (biofermentative) chondroitin in nutraceuticals or pharmaceuticals reduces the environmental impact, since it is obtained by renewable resources, and meets ethical and safety concerns. However, the study of this molecule, although normally present in animal and human tissues, has been addressed only in the recent years, and a full assessment as an active principle needs further in-depth studies. Chondroitin (BC), the unsulfated precursor of CS, obtained from fermentative production processes demonstrated similar or even improved properties in *in vitro* models, as compared to CS. In fact, BC more efficiently reduced the inflammatory response induced by IL-1β and preserved a chondrogenic phenotype (Stellavato et al., 2016), also stimulating the production of type II collagen (Alessio et al., 2021). Russo et al. (2020) analyzed the secretome of synoviocytes treated with fish-extractive CS and biofermentative BC, suggesting not only interesting similarities but also improved activity of BC in the modulation of inflammation biomarkers. BC, alone and combined with HA, was able to reduce NF-kB expression with respect to pathological human chondrocyte synoviocytes (Vassallo et al., 2021). More recently, BC was used in comparison to CS in methacrylated gelatin scaffolds, showing a role in the differentiation of stem cells into chondrocytes (Vassallo et al., 2022).

In this study, BC was evaluated *in vivo* for the first time in mice treated with MIA to resemble pain associated with OA using CS as the control, due to its already established use in the treatment of OA.

The characterization of purified BC and of commercial pharmaceutical-grade CS indicated similar hydrodynamic properties (e.g., MW and polydispersity) and purity, indicating the absence of sulfate groups as the main difference among the two biopolymers. Recent studies suggested that the sulfation pattern and degree of glycosaminoglycans modulate various cellular processes by affecting the interaction with intermediary molecules such as growth factors, adhesion molecules, and lipoproteins (Vlodavsky et al., 1996; Hintze et al., 2014). In fact, different potential pharmaceutical applications of CS, in relation to its sulfation pattern, have been described (Malavaki et al., 2008; Lauder 2009). It was therefore interesting to investigate whether unsulfated chondroitin as well as CS extracted from fish cartilage exhibited anti-osteoarthritic properties.

Among animal models, MIA represents the most used paradigm to study chronic pain associated with OA (Guzman et al., 2003). Along with joint damage, MIA injection triggers mechanical sensitivity in the ipsilateral hind paw lasting at least 1 month. In this study, it was observed that similarly to CS, BC was able to reverse the MIA-mediated mechanical allodynia up to 4 weeks. Interestingly, the analgesic effect was associated with the partial recovery of cartilage loss. In fact, MIA injection has been shown to generate chondrocyte death, neovascularization, subchondral bone necrosis, and collapse, which are reflective of some aspects of patient pathology (Pitcher et. al., 2016). In this study, hematoxylin and eosin staining of representative mouse joints indicated that MIA-mediated morphological changes of the articular cartilage were partially reduced by chondroitin. However, no morphological differences were detected between CS and BC treatments indicating a similar effect. Furthermore, qualitative analyses run as immunofluorescence for COMP-2 selected as a biomarker in pathological joints/cartilage indicated that the MIA group showed an increase in the biomarker, coherently with the damage, that seems to be counteracted by CS and BC treatments, indicating a similar effect. CS was already established by previous scientific reports as a structure-modifying OA drug (SMOAD), and also the *in vitro* functional bioactivity of BC despite the absence of sulfate groups was previously demonstrated (Vassallo et al., 2021).

The potential of chondroitin to counteract the production of mediators known to be harmful for cartilage homeostasis was also investigated. It has been reported that an anti-cytokine treatment may be helpful in the management of OA, reducing its progression and restoring a healthy environment. In fact, pro-inflammatory cytokines play an important role in OA by accelerating synovial inflammation as well as cartilage destruction. IL-1β is considered a key mediator in the progression of OA inflammation (Na et al., 2020). It has been suggested that IL-1β induces an initial response to OA inflammation by upregulating proteins and enzymes involved in cell cartilage damage and downregulating the biosynthesis of proteoglycans and collagen that are the main components of cartilage (Lee et al., 2020). BC efficiently reduced the levels of serum inflammatory cytokines, such as IL-1β, TNF-α, and IFN-ƴ similar to CS. This suggests an improvement of the initial inflammatory response induced by MIA and demonstrates the efficacy/protection of these GAGs against OA inflammation. This mechanism was already observed *in vitro* as reported by Stellavato et al. (2016) and Vassallo et al. (2021) and is confirmed in a more complex animal model of pathological condition. As demonstrated by Vassallo et al. (2021), BC reduces the expression level of NF-kB in a model of human chondrocytes derived from osteoarthritic patients, playing as the modulator for cytokine secretion. These beneficial outcomes may be mediated by receptors. For instance, the action of CS could be mediated by the receptor PTP-sigma (Senis and Bar, 2018). Due to the absence of sulfate groups, BC has a different charge compared to CS, while the repetitive dimer unit is identical to that of CS; therefore, it could be hypothesized that binding to the same receptor occurs. On the other hand, a strong similarity should be recognized with the major unsulfated GAG in nature, hyaluronic acid (HA). In fact, the repetitive disaccharide unit of HA is composed of glucuronic acid, as that of BC, and acetylated glucosamine instead of acetylated galactosamine (present in BC). Thus, further studies are needed to better unravel the biophysical and biochemical features of BC.

The ability of BC to mitigate the inflammation by enrolling Treg cells was also evaluated in this study. Keller et al. (2021) have demonstrated that regulatory T cells induce protection of cartilage in a tri-culture model of osteoarthritis by increasing TIMP1, IL10, and IL4. In the present setting, BC increased the percentage of T regulatory cells in blood, compared to the control. In addition, unexpectedly, BC was more effective than CS in Treg enrichment further indicating that BC, as well as CS, could mitigate the inflammation.

Notwithstanding the structural difference due to the lack of sulfate groups, for the first time, in *in vivo*, BC proved a potentially valid alternative to CS for the treatment of OA. This study in fact demonstrated that BC administration, as that of CS, significantly reduced the severity of OA and mechanical allodynia in MIA-induced osteoarthritic mice. These results were also accompanied by a decrease in inflammation- and pain-related biochemical markers.

## Data Availability

The original contributions presented in the study are included in the article/Supplementary Material, further inquiries can be directed to the corresponding authors.
