# Supplementary material for: Evaluation of unsulfated biotechnological chondroitin in a knee osteoarthritis mouse model as a potential novel functional ingredient in nutraceuticals and pharmaceuticals
Source: Front Bioeng Biotechnol. 2022 Nov 17;10:934997. doi: 10.3389/fbioe.2022.934997 (PMC9714611; doi:10.3389/fbioe.2022.934997)
Supplement: Supplementary file 1 [file DataSheet1.PDF]

Supplementary Figures

A)

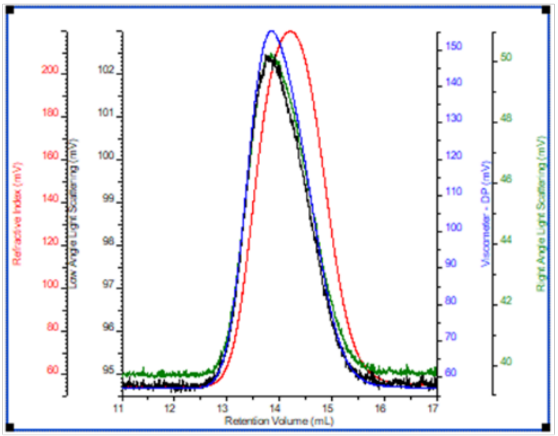

B)

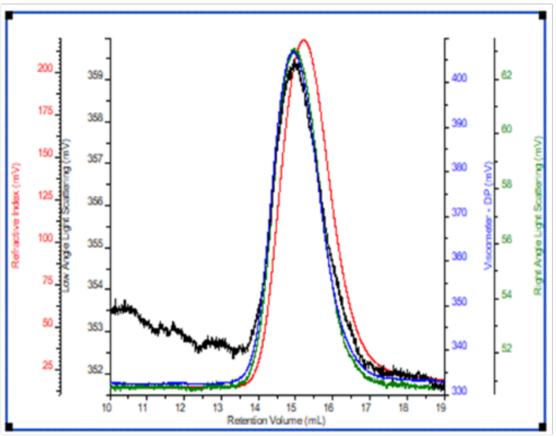

Supplementary Figure 1. SEC-TDA analyses of purified BC (A) and of commercial CS (B). IV, Intrinsic viscosity; Wt Fr, Weight fraction; Rh, Hydrodynamic radius.

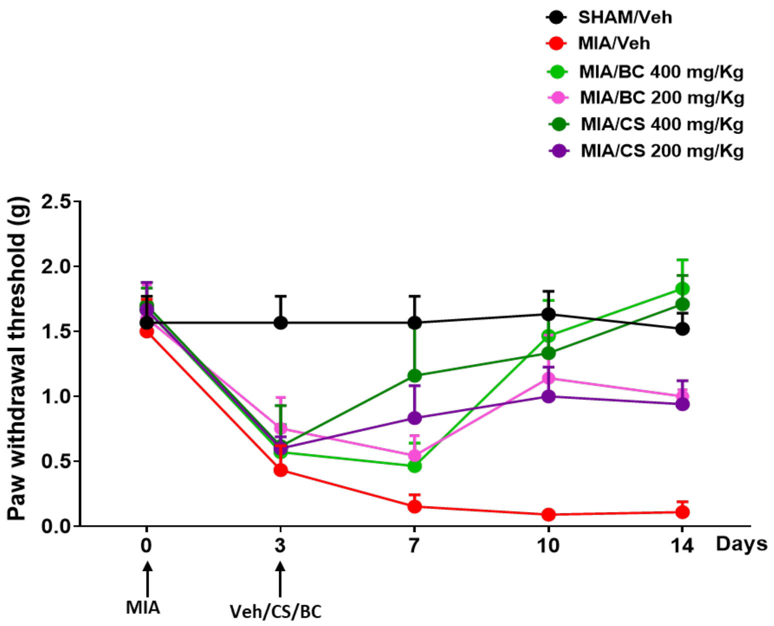

*vs MIA/Veh

	MIA+BC 400 mg/Kg	MIA+BC 200 mg/Kg	MIA+CS 400 mg/Kg	MIA+CS 200 mg/Kg	SHAM/Veh
0	ns	ns	ns	ns	ns
3	ns	ns	ns	ns	**
7	ns	ns	*	ns	***
10	***	**	***	*	****
14	****	*	****	ns	***

Supplementary Figure 2. Effect of daily administration with BC or CS (200 and 400 mg/kg) or vehicle (saline) on the mechanical allodynia in MIA-injected mice. Time-dependent changes in the paw withdrawal response (g) in SHAM and MIA mice treated with vehicle, or BC or CS. *$p<0.05$ **$p<0.01$ ***$p<0.001$ and ****$p<0.0001$ indicate significant differences vs MIA/veh. Two-way Anova, followed Tukey's post hoc.
